# Portugal nutritional transition during the last 4 decades: 1974–2011

**DOI:** 10.1016/j.pbj.0000000000000025

**Published:** 2018-09-05

**Authors:** Alexandra Bento, Carla Gonçalves, Tânia Cordeiro, Maria Daniel Vaz de Almeida

**Affiliations:** a Faculty of Biotechnology, Catholic University of Portugal; b Faculty of Nutrition and Food Science, University of Porto; c Fernando Pessoa University, Porto, Portugal.

**Keywords:** food balance sheet, food trend, Mediterranean diet, nutritional transition

## Abstract

**Objective:**

To examine trends in food availability for Portugal during the last 4 decades (1974–2011) and analyze such changes in accordance with the nutritional transition theory.

**Methods:**

Food balance sheets from Portugal from 1974 to 2011 were analyzed for potential trends by linear regression to study the availability of protein, fat, carbohydrate, ethanol, and total energy and the availability of the following food groups: (i) cereals and tubers; (ii) vegetables; (iii) fruit; (iv) milk; (v) meat, fish, and eggs; (vi) fat; (vii) pulses; (viii) alcoholic beverages; and (ix) sugar and sweeteners. A comparison regarding protein, fat, and carbohydrate availability and WHO recommendation was also performed.

**Results:**

The data suggest that in Portugal food availability and consumption have changed throughout the analyzed period. The national availability of most food groups increased considerably, which also resulted in an increase in daily energy. The consumption of cereals and tubers, pulses, and alcohol diminished during this time. Energy availability increased by 406 kcal/person/day, a result from an increase in protein and fat. Protein availability was in accordance with WHO recommendations during the 4 decades analyzed, whereas carbohydrate have always been below the recommended level and fat has been above the recommended level since the second decade (1984).

**Conclusion:**

Portugal has crossed into a nutritional transition over the last 4 decades, revealing characteristics of a pattern of degenerative diseases. The country may experience a new nutritional transition that would involve positive changes of behavior, as observed in other developed countries, driven by community multisectorial strategies.

## Introduction

Epidemiological transition has been defined as the combination of the demographic transition model with changing patterns of mortality, fertility, life expectancy, and causes of death. The last stage of this model has been characterized by an aging population and high mortality rates attributable to cardiac and cerebrovascular ailments, chronic lung and metabolic diseases, and cancers, that is, the major causes of death were noncommunicable diseases (NCDs).^1^

Over time, populations worldwide have undergone a series of nutritional transitions, in parallel to the country's economic development usually followed by improved food availability. The concept of nutritional transition integrates the process of demographic and epidemiological transition. Nutritional transition is a process of sequential modifications in the pattern of nutrition and consumption, which accompanies economic, social, and demographic changes, and changes of the health profile of a population.^2^ Although the literature focuses more on the Westernization of diets, which involves the increased consumption of meat, fat, processed food, sugar, and salt, Popkin^3^ described 5 patterns that can be observed in various societies:

1. *Collecting food pattern*. This diet is high in carbohydrates and fiber and low in fat, especially saturated fat, characterized by plants and wild animal's intake.
2. *Famine pattern*. The diet becomes much less varied and is subject to larger variations and periods of acute scarcity of food.
3. *Receding famine pattern*. The consumption of fruits, vegetables, and animal protein increases, and starchy staples become less important in the diet.
4. *Degenerative diseases pattern*. A diet high in total fat (especially from animal products), sugar, and other refined carbohydrates and low in fiber often accompanied by increasingly sedentary life, resulting in increased prevalence of obesity and contributing to the degenerative diseases.
5. *Behavioral change*. This dietary pattern is associated with the desire to prevent or delay degenerative diseases and prolong health, characterized by less fat and processing foods and increased carbohydrates, fruits, and vegetables intake.

In the present work, the nutritional transition of a developed southern European country with a temperate climate, located at the intersection of the Atlantic Ocean and the Mediterranean Sea was analyzed for the last 4 decades. Portugal, like Spain, was not as deeply affected as other parts of Mediterranean Europe by World War II.^4^ It was, however, affected by 48 consecutive years of presidentialist, authoritarian, and antiparliamentary conceptions of government (1926–1974). During this period, Portugal has remained isolated from the Europeanist spirit, maintained minimal social development, and faced the Colonial War between 1961 and 1974. After the 1974 revolution, Portugal implemented a democratic political system, joined the European Union, and experienced rapid social change.

Since 1974, Portugal has crossed a demographic transition shift, from a pattern of high fertility and high mortality to 1 of low fertility and low mortality with an increased in life expectancy (from 68.2 years in 1974 to 80.2 years in 2013).^5^ This epidemiologic transition, most relevantly, also marked a change from a pattern of high prevalence of infectious diseases to a high prevalence of chronic ones. In 2012, almost 86% of deaths were due to NCDs, of which 32% were from cardiovascular diseases, 28% from cancer, 6% chronic respiratory diseases, and 5% from diabetes mellitus.^6^ Furthermore, the phenomena of demographic and epidemiological transitions, reflected by the double aging of the Portuguese population, that is, a smaller proportion of young people and an increase in the elderly population, were associated with the epidemiological transition that placed the NCD in the top categories, both in terms of morbidity and mortality.^7^

The main aim of this article is to investigate trends in food availability for Portugal during the last 4 decades (1974–2011), and to analyze the findings in accordance with the nutritional transition theory.^3^

## Methods

Data were obtained from the Food Balance Sheet (FBS) published by the FAO (database: FAOSTAT).^8^ The FBS allows to estimate the annual food availability of a country, showing estimates about the quantities and groups of the main foodstuffs available for human consumption. Therefore, and as in similar works, the expressions “food consumption” or “food intake” should be understood as food available for consumption, or as an apparent average consumption.^9^

Data about food availability concerning equivalent primary products such as wheat, maize, potato, rice, other cereals, other tubers, milk, vegetables, fruit, beef, pork, poultry, other meats, fish, molluscs and crustaceans, pulses, vegetable oils, olive oil, butter, cream, lard, fish oils, sugar, sweeteners, honey, wine, beer, and other alcoholic beverages, which are later aggregated into the following groups according to the Portuguese food guide: cereals and tubers; vegetables; fruit; milk; meat, fish, and eggs; fats; and pulses. In addition, the groups of alcoholic beverages, sugars, and sweeteners were also analyzed.

Data about nutrient availability (protein, lipid, and ethanol in kg/person/year) and energy availability (kcal/person/day) were obtained. In order to calculate carbohydrate availability we subtracted ethanol, protein, and lipids to the total daily energy (kcal/person/day) and converted the value into grams, considering the Atwater coefficients that 1 g of carbohydrate equals 4 kcal. Subsequently food data were converted to g/person/day for analysis.

Data on trends in food and nutritional availability are related to the last 40 years, so data from period between 1974 and 2011 (the most recent data available) were collected from the FAO database. In order to group the analyzed decades under observation, the following periods were formed: period I (years 1974–1983), period II (1984–1993), period III (1994–2003), and period IV (2004–2011). The last period only contemplates 8 years, due to the unavailability of more recent data.

Subsequently, the average variation of energy and nutrient availability (percentage of total energy value) between periods I, II, III, and IV and comparison of mean values in each period with values recommended by the WHO were performed.^9^

### Statistics

The trend over time was analyzed using linear regression models. Trends are described by straight segments but where changes are allowed over the study period. The year in which a change in trend is detected throughout the study period is called a “breakpoint” and significant breakpoints are identified.^10^

In this study, the number of breakpoints was defined according to the Bayesian Information Criterion. Starting at 0 breakpoints and increasing 1 breakpoint at each step, the best solution is identified when increasing the number of breakpoints does not imply a decrease in the Bayesian Information Criterion. This analysis was used to study the evolution of nutritional availability using software R version 3.0.1.

## Results

In Table 1 we can verify the evolution in the availability of food groups according to breakpoints from 1974 to 2011. Regarding vegetables and pulses, no points of change were identified; however, in case of vegetables there was an increasing availability and in case of pulses there was a decrease in availability. The food groups that showed more points of change during the period analyzed were cereals and tubers and the group of meat, fish, and eggs, where 5 points of change was identified.

**Table 1:**
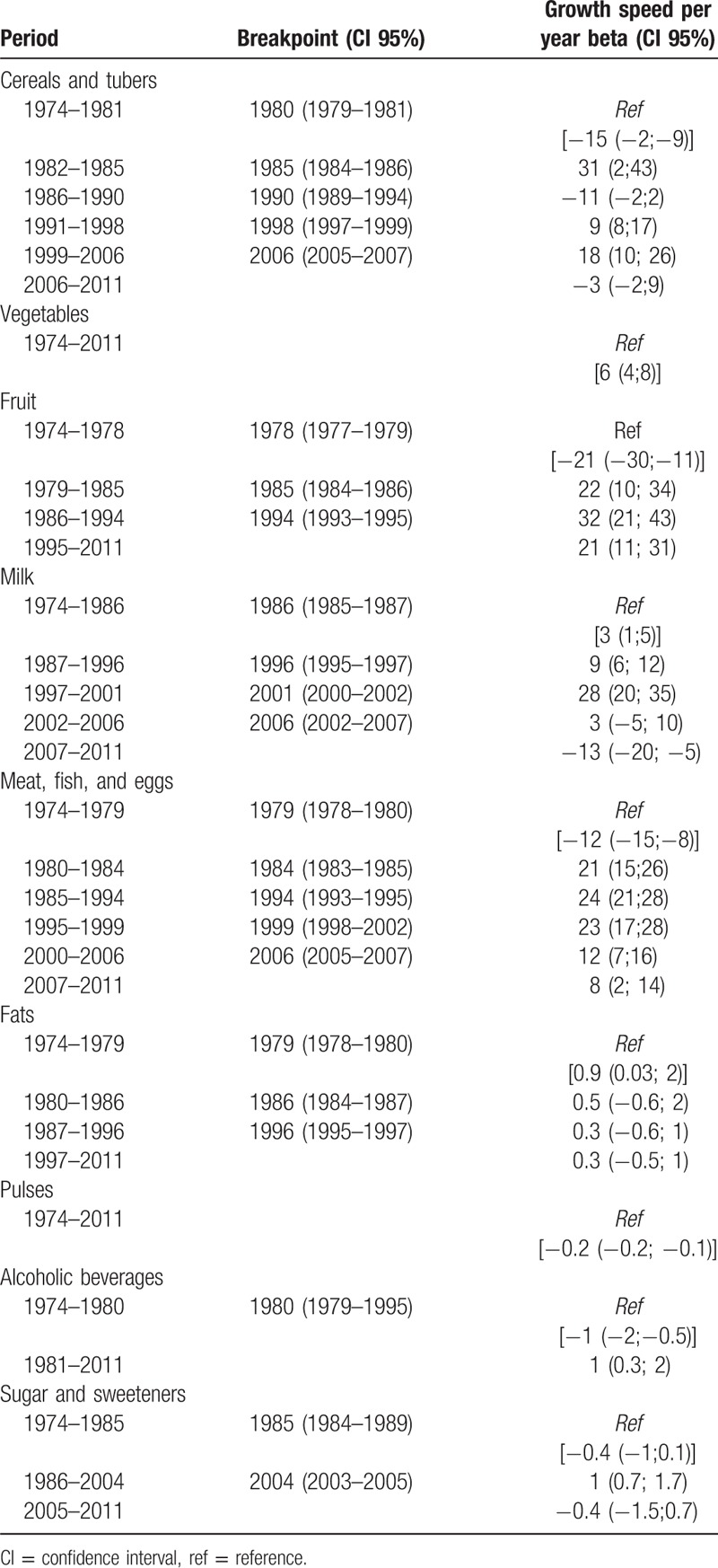
Evolution of the availability of cereals and tubers; vegetables; fruit; milk; meat, fish, and eggs; fat; pulses; alcoholic beverages; and sugar and sweeteners according to the “breakpoints” from 1974 to 2011

Table 2 shows trends in the availability of energy, macronutrients, and ethanol between 1974 and 2011. In relation to the evolution of energy availability, 3 breakpoints are identified: between 1974 and 1981 availability decreased (annual speed = 47 g/person/day); between 1982 and 1986 availability increased (annual speed = 83 g/person/day); between 1987 and 2003 increased at a slower speed (annual speed = 14 g/person/day); and in the last period between 2004 and 2011 there was a decrease in availability (annual speed = 17 g/person/day). In the case of ethanol availability, only 1 breakpoint is found during the analysis period, the availability decreased until 1980 (annual speed = 1 g/person/day) and thereafter stabilized. Protein was the macronutrient that showed more breakpoints, there were 4 breakpoints: until 1980 decreased (annual speed = 2 g/person/day) and after that there was an increase at different speeds.

**Table 2:**
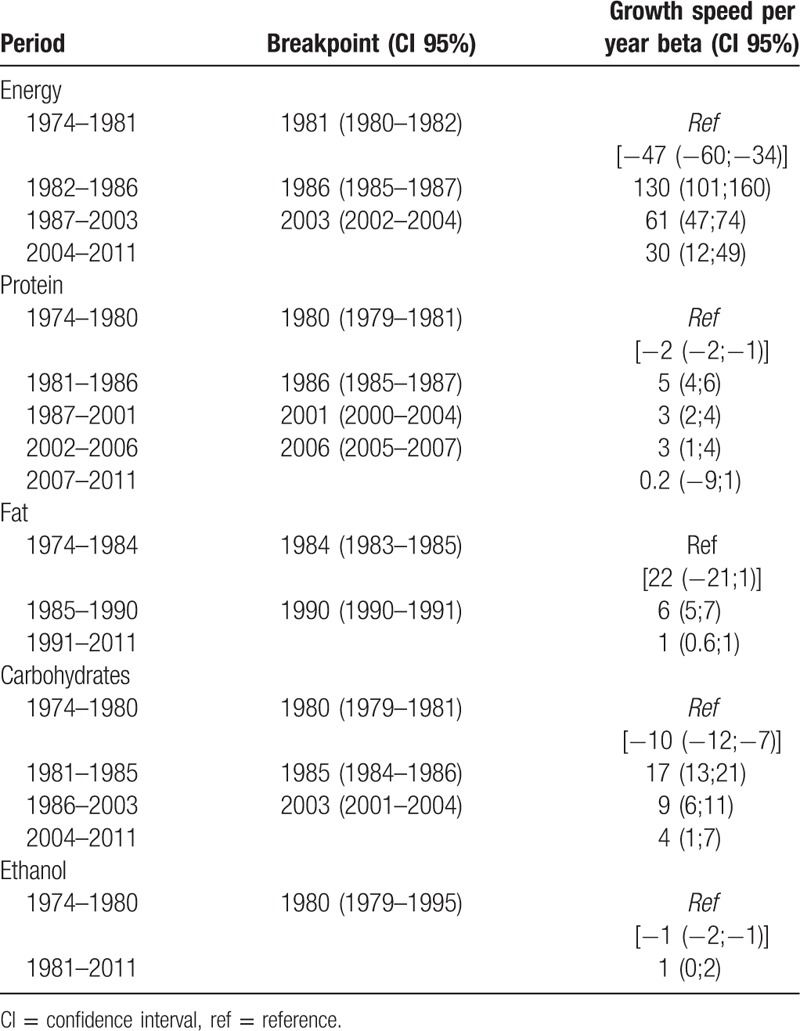
Evolution of the availability of energy, protein, fat, carbohydrates, and ethanol according to the “breakpoints” from 1974 to 2011

Over time, the mean available energy (kcal) increased progressively from decade to decade, with a mean increase of 669.8 kcal/person/day from 1974 to 2011. The same is true for the energy contribution of protein and lipids (increase 1.9% and 8.0%, respectively). On the contrary, there was a decrease in the energy contribution of carbohydrate (6.7%). Comparing these values with the recommendations for the adequate intake of macronutrients from the WHO (Table 3), it is verified that the energy contribution of the protein is in line with the recommendations; the energy contribution of the carbohydrates is below the recommendations and fat is above recommendations since period II.

**Table 3:**
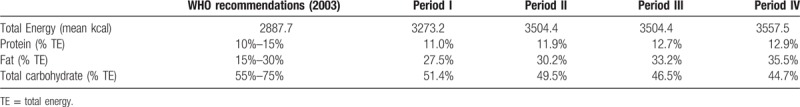
Comparison of the WHO recommendations with the availability of the macronutrients in period I (1974–1983), period II (1984–1993), period III (1994–2003), and period IV (2004–2011)

## Discussion

In Portugal, as in other countries,^9,11,12^ the use of FBS over the years allows time trends analysis in food availabilitywhich in combination with health indicators sheds light on the positive or negative features of food patterns. In recent decades, economic development, increased purchasing power, innovation in food production, and food globalization have changed the food and nutritional situation, with changes in food availability and consequent changes in the health profile of countries, namely nontransmissible chronic disease prevalence.^9,13,14^

The analyzed data show that the Portuguese have changed their food profile throughout these 4 decades. It was found that the national consumption of most food groups increased considerably (milk group; meat, fish, and eggs group; fruit group; fats group; sugar and sweetener group; and vegetable group) and consequently resulted in an increase in daily energy consumption. The consumption of some foods, however, decreased in this time, such as cereals and tubers, pulses, and alcoholic beverages. This trend was similar to that found in global studies of developed countries^12^; therefore, Portugal can be considered a developed country when compared to the described food standards.^9^

It is possible to verify that the years 1978 and 1979 marked an increase in the consumption of fruit; meat, fish, and eggs; and fats, and later, the years 1985 and 1986 marked an increase of the consumption of sugars and sweeteners and milk. These milestones can be analyzed in light of the political, social, and economic evolution of the history of Portugal.

From 1933 to April of 1974, an authoritarian, autocratic and corporatist state political regime presided over Portugal, allowing for minimal social development. The period between 1961 and the 1974 also included the Colonial War, which involved large expenditures by the State to withstand the conflict. After 1974, a complex process of political and social organization took place. Following the Revolution, Portugal sought to promote its presence in the European Free Trade Area Association until 1977 and submitted its application to the European Economic Community (EEC), which was accepted in 1986.^15^ This marked a greater availability of food (due to food importation) and the receival of economic funds, improving the socioeconomic conditions of the country. Since joining the EEC, domestic agricultural production has grown less than food supply, resulting in a growing food deficit.^16^

The 1990s can be described as years of wealth and economic growth, and hence the consumption of sugar and sweeteners, milk and meat products, and fish and eggs has drastically increased.

In 2002, the Portuguese economy entered a phase of stagnation or recession with high unemployment rates, culminating in a request for a financial rescue to the European Commission in 2011. This phase coincided with the stabilization/decrease in the consumption of various foods such as cereals and tubers (from 2006); milk (from 2007); meat, fish, and eggs (stabilized between 2000 and 2006 and reduced from 2007); and sugars and sweeteners (from 2005).

The picture described justifies the need for reflection on variations in the availability of each food group and nutritional and energy availability.

The decline in the availability of cereals and tubers is in accordance with trends of other developed countries whose cereal availability has also declined.^17–19^ In a systematic analysis of 113 countries, it was found that cereal consumption decreased by 8.5 g/day between 1990 and 2010, a reduction that also occurred in Europe.^12^ In addition, Garcia-Closas et al^17^ found that there was a decline in the availability of cereals in Europe during the period 1961 to 2001, in addition to an increase in Africa and Asia. In Spain, an analysis from 1964 to 1991 showed a reduction in cereal consumption to about half.^4^ The availability of cereals and tubers had the highest number of break points during the study period, with periods of decreased availability interwoven with periods of increased availability (their most visible breakpoints were essentially related to the availability of wheat and potatoes). Wheat has been increasing in availability and contributed most to this food group in 2011, surpassing the availability of potatoes that, by 1998 had provided the most significant contribution.

The consumption of fruits and vegetables sharply increased between 1974 and 2011. The availability of fruits and vegetables was found to be above the WHO recommended level,^20^ representing approximately 568 g/person/day in 1974, and approximately 767 g/person/day in 2011; the consumption of fruits (increased by 115.6 g/person/day) had increased more than that of vegetables (up 83.6 g/person/day). These data are consistent with the situation in other countries, particularly in Mediterranean countries.^9,17,21^

In Portugal, milk consumption increased from 1974 to 2011. However, since 2007, there has been a decrease in consumption. In 1986, the year Portugal joined the Common Agricultural Policy, and benefit from EEC funding, so there was a substantial increase in the production and importation of milk, thus, increasing its availability. This increase in consumption has also been mentioned in studies with data from the National Health Survey.^22^ In 1991, the milk quota was fixed in Portugal, and in 2002/2003 and 2005/2006, Portugal even had to pay a fine for excess production. The increase in milk consumption was also observed in most Mediterranean countries, with an impact on the increase in energy consumption from animal fat.^17^ From 2007, milk consumption decreased, and the consumption of cereal and pulses sugary drinks (vegetable drinks popularly denominated as vegetable milk) in substitution of milk consumption increased, both of which were associated with the onset of the economic crisis. These beverages are often associated, by public opinion, with a healthier lifestyle, and their consumption is justified by arguments with fragile scientific support.

The group that has contributed most to protein consumption since 1989 is meat, fish, and eggs, which had previously been cereals. This inversion is often pointed out as a key moment of nutritional transition, which leads us to reflect about the case of Portugal.^2,3,23,24^

The first nutritional transition occurred in high-income countries and in developing countries, in which sustenance became rich in fat, sugar, and processed foods, and low in fiber, leading to an increase in NCD. In their work, Vranken et al^25^ pointed out that a further transition to a fifth pattern may be due to a change motivated by increased knowledge of environmental concern, health, and consumer behavior, and the reduction of meat consumption is often pointed out as one of the characteristics of this change.

The present work shows that meat availability more than doubled between 1974 and 2011, with this increase having been verified with a greater increase in the availability of pork and poultry. In 1992, this type of meat became more available than beef. The decrease in the availability of beef may have been due to the impact of the bovine spongiform encephalopathy crisis.^26^ According to this logic, the production and availability of pork and poultry will have benefited from this space in the market. Furthermore, the fact that the livestock of these animals produces a lower environmental impact than the production of cattle has also been pointed out as a determining factor for this phenomenon.

In relation to the availability of fish, it was possible to observe that, although this availability had undergone several oscillations throughout the determined period, it became relatively stable from 1985, with a slight progressive diminution.

The Mediterranean Diet advocates the consumption of fish rich in polyunsaturated fatty acids (n-3) with a frequency of 4 to 5 servings per week.^27,28^ Portugal is considered to have several features a Mediterranean country, due to climate and nature, agrofood production, conviviality, and sociability, as well as typical Mediterranean cuisine.^29,30^

The geographical location of Portugal, with an extensive coastline conducive to the development of maritime activities, favored the early establishment of communities in which fishing was the main labor activity.^31^ This activity showed an increasing trend until the mid-1960s, and a decrease has been observed since then.^31–33^ Associated with this intense activity and the availability of fish, especially in the coastal zone, the habit of fish consumption in Portugal has become favorable. According to data from FAO, Portugal had the highest fish consumption in 2005, in comparison to other EU countries.^34^

The decline in fish consumption, coupled with an increase in meat consumption, could lead to the idea of divergence from the Mediterranean diet. The decrease in fish consumption could be due to the influence of Western eating habits, the globalization of production and consumption, the lower availability of fish, and the unavailability of processed fish products with easy preparation, as a response to the current lifestyle.^35–37^

One of the characteristics of the nutritional transition to the typical pattern of NCDs defined by Popkin^3^ is the increase in fat consumption, which has been seen in several other countries throughout the world.^9^ This trend of increasing fat consumption has also been consistently observed in Portugal from 1974 to 2011, with an increase in the consumption of vegetable and animal fats (note that the consumption of animal fats increased significantly between 1974 and 2011, as the values almost tripled).

In 1974, the consumption pointed to the main sources of fats, vegetable oils, and olive oil; however, in 2011, vegetable oils and lard contributed most to the consumption of fats. Vegetable oils continue to be the food that contributes most to the fats group, although there has been a significant decrease between 1988 and 1996. Fats of animal origin, butter and lard, contributed most to the increase in the availability of fats from 1974 to 2011. Although olive oil plays an important role as the fat of preference for the Mediterranean diet,^38^ its consumption has declined since 1974 until 1984, when its availability was less than the availability of lard. There was, however, a significant increase in the availability of olive oil from 2007 to 2011. Portugal is one of the highest consumers of olive oil in Europe.^39^ As can be seen in numerous studies worldwide, the trend of the availability of olive oil varies based on the decade under study. Between 1966 and 2003, the availability of olive oil decreased, and that of vegetable oils increased in Portugal, with a similar trend in other Mediterranean countries, in which a reduction in the contribution of olive oil compared to that of other vegetable oils.^40^ The significant increase in the consumption of olive oil in Portugal in the last decade may be related to health professionals’ awareness of its health benefits and the consequent recommendations toward its consumption,^39,41^ and may have also been due to the improvement of the socioeconomic conditions of the population.

As for the consumption of pulses, this gradually declined from 1974 to 2011. As the Mediterranean diet is traditionally rich in pulses,^42^ the gradual decline in its consumption may also have resulted from changes in consumption patterns and consumer preferences^43^ to a possible Westernization of consumption, in which these foods do not play a prominent role.

In relation to the consumption of alcoholic beverages, we verified that this decreased during the period studied. Furthermore, in 1988, there was a reversal of the consumption pattern, as having beer became the most consumed beverage, over wine. Looking more closely at this phenomenon, the increasing beer availability may explain the observed decline in ethanol consumption.

Wine has an average ethanol content of half than that of beer.^44^ A study of ethanol consumption published by Balsa et al^45^ found that, in Portugal, wine consumption has accompanied various social, demographic, political, and economic changes verified over time. The increase in purchasing power after the 1970s, coupled with the liberalization of customs and the greater aggressiveness of breweries and companies selling distilled spirits, could be the basis of the change in the pattern of consumption and the induction of beer consumption among young people and women.^46^

The consumption of alcoholic beverages in Portugal should be understood as a social act inserted in a context of values, religion, attitudes, and norms, deeply rooted in a wine country, as the rest of the Mediterranean countries.^46,47^ The consumption of alcoholic beverages, and consequently, of ethanol, may, however, constitute a social and public health problem when it occurs in situations that may simultaneously interfere with family, school, occupational, and social life of the consumer, leading to associated addiction problems.^45^

The Westernization of food consumption, already mentioned above, can also be characterized by an increase in the consumption of sugar and sweeteners,^48^ an increasing trend in several countries since the 1960s.^49^ Furthermore, in Portugal, the consumption of sugars and sweeteners increased until 2005, and subsequently decreased. A similar trend was observed in the United States of America, where sugar consumption increased until the mid-1990s, and began to decline from the beginning of the 21st century; this reduction was associated with a reduction in the consumption of soft drinks.^50^ Between 1974 and 1983, sugar contributed to 9% of the total energy value; this contribution decreased in the remaining years, ending in 2004 to 2011 with a contribution of 7.7% of the total energy value. Although this value is lower than the WHO recommended level of a maximum of 10% of total energy value, a 5% target should be considered in policy making due to its health benefits.^51^

In the period under study, energy consumption increased by 406 kcal/person/day, a picture similar to the average world energy consumption.^9^ In developing countries, this increase has occurred at a fast speed.^9^ The availability of energy began to increase at a rate of 47 g/person/day in 1982, a time that coincided with the increase in the availability of various foods, and began to decrease in 2004, the beginning of the “economic crisis” in Portugal.

The origin of energy over the period under review has changed, with an increase in animal sources. A more detailed analysis showed that the drivers of this change were animal proteins and, more markedly, lipids of animal origin (data may be related to the previously analyzed increase in consumption of meat, fish, eggs, and milk analyzed previously).

The increase in lipids consumption was progressive. Between 1974 and 1983, lipids consumption in Portugal was in accordance with WHO recommendations,^9^ and it grew to a consumption that exceeded the same (+5.5%). The food groups that contributed most to this energy increase were fats and meat, fish, and eggs.

The analysis of the protein showed that this nutrient presented more breakpoints in the analyzed period, and was characterized by a strong increase of availability between 1981 and 2006, the year in which it began to decrease. Despite fluctuations in availability, protein consumption remained in accordance with the WHO recommendations.^9^

From the analysis of the evolution of carbohydrates availability, it was verified that an increase occurred in the years 1981 to 1985, and the consumption decreased in the remaining years. Despite the fluctuations, the contribution of carbohydrates to total energy has always remained below the WHO recommendations,^9^ and is explained by the availability of the amount of energy coming from the cereal group. An analysis of world consumption shows that the contribution of the cereals group has been felt in developing countries, such as China and Brazil, mainly at the expense of reducing the consumption of wheat and rice.^9^ The changes in the energy contribution of the cereals group in Portugal are more related to the variation in the consumption of potatoes, not wheat and rice.

According to the definition of nutritional transition by Popkin,^3^ and taking into account the analysis of the trends in food consumption, we can consider that Portugal is crossing into the fourth pattern, the degenerative diseases pattern, especially due to an increase in the availability in total fat from animal products and in sugar, during the last decades. The country may progress to the fifth pattern, largely due to reduction on meat consumption, as described by Vranken et al.^25^ To this, policy makers need to develop environmental and health policies that provide opportunities for positive change of food behavior of citizens, including the promotion of Mediterranean diet as a healthy pattern.^36^ The design of a national nutritional policy should be evidence-based and follow the international guidelines proposed by authorities, proposing a cross-sectoral mix of interventions to ensure physical and economic access to healthy eating by creating healthy environments and empowering individuals and communities.^13^

It should be noted that FBS data could form the basis for a national food consumption monitoring system in a country without an established national food policy and without a vigilance system of food consumption. However, the establishment of food consumption monitoring and surveillance systems with greater detail and accuracy of data will be of great importance for the establishment of food and nutrition intervention programs as well as for the definition of a national food policy focusing on the prevention of chronic diseases and the maintenance of health.

## Conclusion

Over the last 4 decades, Portugal witnessed a nutritional transition that is characterized by increased life expectancy, with a predominance of NCDs related to diet. The country could cross into a new nutritional transition that will entail a positive change of behavior, as observed in other developed countries. These changes ought to be driven by community multisectoral strategies as well as preventive measures developed within the framework of a national nutritional policy.

## Acknowledgments

The authors acknowledge the statistical consultation of statistician Milton Severo (PhD) and epidemiologist Carla Lopes (PhD) of the Institute of Public Health of the University of Porto.

## Conflicts of interest

The authors report no conflicts of interest.
